# A novel role for Ascl1 in the regulation of mesendoderm formation via HDAC-dependent antagonism of VegT

**DOI:** 10.1242/dev.126292

**Published:** 2016-02-01

**Authors:** Li Gao, Xuechen Zhu, Geng Chen, Xin Ma, Yan Zhang, Aftab A. Khand, Huijuan Shi, Fei Gu, Hao Lin, Yuemeng Chen, Haiyan Zhang, Lei He, Qinghua Tao

**Affiliations:** 1 MOE Key Laboratory of Protein Sciences, Tsinghua University School of Life Sciences, Beijing 100084, China; 2 CAS Key Laboratory of Genome Sciences and Information, Beijing Institute of Genomics, Chinese Academy of Sciences, Beijing 100101, China; 3 Tianjin Normal University College of Life Science, Binshuixidao (extension line) 393, Xinqing District, Tianjin 300387, China

**Keywords:** Ascl1, Transcriptional repressor, HDAC1, Mesendoderm, VegT, *Xenopus*

## Abstract

Maternally expressed proteins function in vertebrates to establish the major body axes of the embryo and to establish a pre-pattern that sets the stage for later-acting zygotic signals. This pre-patterning drives the propensity of *Xenopus* animal cap cells to adopt neural fates under various experimental conditions. Previous studies found that the maternally expressed transcription factor, encoded by the *Xenopus* achaete scute-like gene *ascl1*, is enriched at the animal pole. Asc1l is a bHLH protein involved in neural development, but its maternal function has not been studied. Here, we performed a series of gain- and loss-of-function experiments on maternal *ascl1*, and present three novel findings. First, Ascl1 is a repressor of mesendoderm induced by VegT, but not of Nodal-induced mesendoderm. Second, a previously uncharacterized N-terminal domain of Ascl1 interacts with HDAC1 to inhibit mesendoderm gene expression. This N-terminal domain is dispensable for its neurogenic function, indicating that Ascl1 acts by different mechanisms at different times. Ascl1-mediated repression of mesendoderm genes was dependent on HDAC activity and accompanied by histone deacetylation in the promoter regions of VegT targets. Finally, maternal Ascl1 is required for animal cap cells to retain their competence to adopt neural fates. These results establish maternal Asc1l as a key factor in establishing pre-patterning of the early embryo, acting in opposition to VegT and biasing the animal pole to adopt neural fates. The data presented here significantly extend our understanding of early embryonic pattern formation.

## Abstract (precis)

**Summary:** The proneural factor ASCL1 recruits HDAC1 to repress VegT-induced, but not Nodal-induced, mesendoderm formation via a previously uncharacterized N-terminal domain.

## INTRODUCTION

Maternal factors play essential roles in coordinating embryonic cell fates in time and space. In *Xenopus laevis*, VegT and Wnt11b (formerly known as Wnt11) represent two distinct types of maternal activities that are essential for early pattern formation (Heasman, 2006). VegT pre-patterns mesendoderm in the subequatorial zone, whereas Wnt11b initiates a β-catenin dependent signalling pathway to establish the embryonic dorsal-ventral asymmetry (Cha et al., 2008; Tao et al., 2005; Zhang et al., 1998). Studies of the regulation of signalling through VegT and Wnt11b pathways have advanced our understanding of molecular mechanisms underlying early vertebrate embryogenesis.

VegT encodes a transcription factor in the T-box gene family (Horb and Thomsen, 1997; Lustig et al., 1996; Stennard et al., 1996; Zhang and King, 1996). VegT activates expression of the zygotic mesendoderm inducers in the TGF-β/Nodal family and a list of conserved mesendodermal lineage genes such as Mix-like factors (Heasman, 2006; Taverner et al., 2005; Xanthos et al., 2001; Yasuo and Lemaire, 1999). The control of the Nodal/Activin signalling activity in various contexts including germ layer pattern formation has been intensively studied (Massague and Chen, 2000; Rogers and Schier, 2011; Tian and Meng, 2006). However, limited information is available regarding how the maternal VegT function is regulated as a principal mesendoderm patterning factor (Cao, 2013; Heasman, 2006).

Vegetal localization of maternal *vegt* mRNA provides a means of controlling its activity in space, thus pre-patterning the primary germ layers along the animal vegetal axis. Cells in the animal pole are pluripotent during the blastula through the early gastrula stages, after which they are committed to *sox2*-expressing neural plate or epidermis under the inductive signals from the organizer (De Robertis and Kuroda, 2004). POU-V factors, which are homologs of Oct3/4 in *Xenopus*, control the pluripotency of embryonic cells and inhibit differentiation induced by signalling through Nodal/Activin, Wnt and VegT pathways (Cao et al., 2007, 2006, 2008; Snir et al., 2006). Intriguingly, *Xenopus* animal pole cells, like epiblast stem cells in mammals, intrinsically tend to adopt neural cell fates (Artus and Chazaud, 2014; Kuroda et al., 2005; Levine and Brivanlou, 2007; Stern, 2005; Torres-Padilla and Chambers, 2014). Moreover, an early study has shown that some neural genes are expressed in VegT-depleted embryos (Zhang et al., 1998). The maternal factors that render the neural tendency of prospective ectoderm are less clear. In light of these early findings, we searched for genes enriched at the animal pole that may be involved in controlling both neural cell fates and VegT-mediated mesendoderm induction.

Vertebrate ASCL1 is orthologous to the bHLH factors encoded by *Drosophila* achaete-scute complex genes (Johnson et al., 1990). ASCL1 is essential for neurogenesis in invertebrates and vertebrates (Bertrand et al., 2002; Castro and Guillemot, 2011; Wilkinson et al., 2013). ASCL1 alone, or together with other factors, converts non-neural somatic cells into neurons (Amamoto and Arlotta, 2014). ASCL1 has also been implicated in cancerous phenotypes of several types of carcinoma (Huang et al., 2014; Jiang et al., 2009; Rheinbay et al., 2013; Wylie et al., 2015). In this study, we provide evidence that *ascl1* is a maternal gene enriched in the animal pole. Both gain- and loss-of-function analyses reveal that Ascl1 is a crucial repressor of mesendoderm and a pre-pattern factor for neural fate. Ascl1, through a previously uncharacterized N-terminal domain, antagonizes VegT function during mesendoderm formation in a HDAC-activity-dependent fashion. Overall, our findings highlight that ASCL1 is a dual function gene essential for early embryonic cell fate specification.

## RESULTS

### Maternal expression of *ascl1* in *Xenopus laevis*

**Fig. 1.**
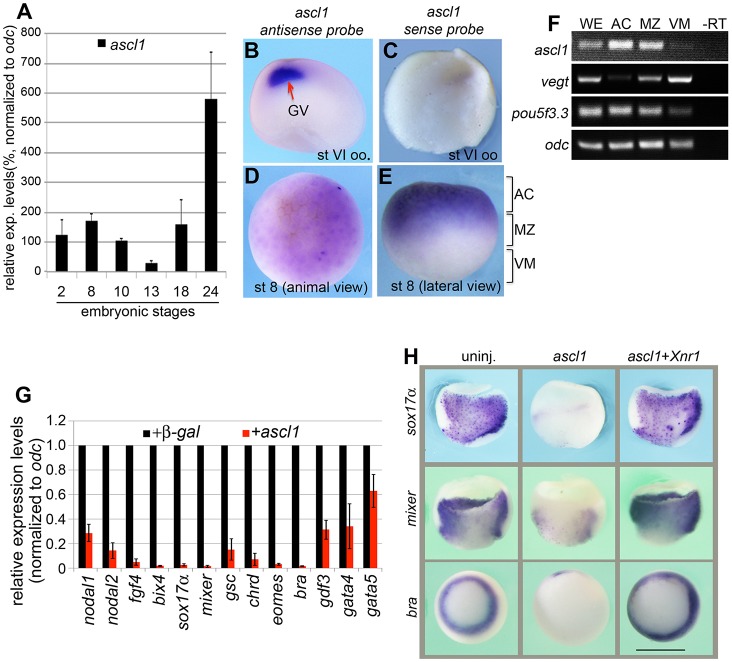
***ascl1* is a maternal gene in *Xenopus* embryos and ectopic ASCL1 represses mesendoderm formation.** (A) Expression of *ascl1* at different stages of *Xenopus* development as analysed by qPCR and represented as the relative expression levels (means±s.d.) to that at stage 10 after being normalized to *odc*. (B-E) Whole-mount *in situ* hybridization for *ascl1* in oocytes and embryos at maternal stages of development. (B) Bisection view of a full-grown oocyte at stage (st) VI. GV, germinal vesicle. (C) Bisection view of a full-grown st VI oocyte *in situ* hybridized with *ascl1* sense probe. (D,E) Animal (D) or lateral (E) view of an embryo at the mid-blastula stage (st8) *in situ* hybridized with *ascl1*. (F) RT-PCR analysis of the relative distribution of *ascl1*, *vegt* and *pou5f3.3* along the animal-vegetal axis of blastulae at stage 8.5, as indicated in E. AC, animal cap; MZ, marginal zone; VM, vegetal mass. (G) qPCR comparison of gene expression in animal cap explants injected with VegT (300 pg) and mRNA encoding β-gal or Ascl1 (500 pg) in the animal pole at the two-cell stage. Animal caps were dissected at stage 8.5 followed by qPCR analysis at the sibling stage 10.5. The expression level of each individual gene (mean±s.d.) was normalized to *odc*. (H) *ascl1* (500 pg) was injected into the vegetal pole, followed by injection of *Xnr1* (100 pg). Expression of mesendoderm genes was analysed by WISH at stage 10.5 (vegetal view). Scale bar: 1 mm.

Previous studies have indicated that Ascl1 is maternally expressed in *Xenopus* (Ferreiro et al., 1994; Collart et al., 2014). However, its expression pattern and function in preneurula development have remained unclear. To understand its maternal function, we confirmed its maternal expression by RT-qPCR and whole-mount *in situ* hybridization (WISH). *ascl1* was persistently expressed in the fertilized egg (stage 2) to the early gastrula stage (stage 10) as shown by qPCR (Fig. 1A). Interestingly, *ascl1* was stored in the germinal vesicle of full-grown oocytes as revealed by WISH (Fig. 1B). A sense probe that was used to control for the specificity did not detect an obvious signal in the germinal vesicle (GV, Fig. 1C), indicating that the *ascl1 in situ* signals detected in the GV are specific. At the mid-blastula stage, *ascl1* was detected in the animal hemisphere by WISH (Fig. 1D,E), forming a distribution pattern complementary to that of *vegt*. Additional RT-PCR analysis with separated animal caps (ACs), marginal zones (MZs) and vegetal masses (VMs) from stage 8 embryos confirmed that both *vegt* and *ascl1* were detected in the marginal zones (Fig. 1F), implying a potential functional interaction between these maternal factors during primary germ layer induction. We found that both alleles of the *Xenopus ascl1* gene, i.e. *ascl1a* and *ascl1b*, are expressed in oocytes and early embryos (data not shown).

### Ectopic ASCL1 inhibits mesendoderm formation

Ascl1 has been appreciated as a major regulator of neural development in invertebrates and vertebrates. It is able to convert mouse embryonic fibroblasts into neurons, during which ASCL1 inhibits mesodermal programs (Wapinski et al., 2013). We therefore asked whether Ascl1 in *Xenopus* represses mesendoderm formation. To answer this, we microinjected synthetic mRNA encoding *Xenopus* Ascl1a, Ascl1b or human ASCL1 into subequatorial region at the two-cell stage. Overexpression of each of these three Ascl1 isoforms inhibited blastopore lip formation and *sox17a* expression (Fig. S1A,B). qPCR examination revealed that ectopic ASCL1 diminished expression of a number of representative mesendoderm genes induced by VegT, as shown in Fig. 1G, but did not affect the expression of maternal *vegt* mRNA (Fig. S1C), suggesting that ASCL1 is sufficient to suppress mesendoderm formation in *Xenopus*. In addition, we performed a series of analyses and determined that ASCL1 specifically inhibits VegT- but not Nodal/Activin-mediated mesendoderm induction. First, coinjection of *ascl1* inhibited the ability of Myc-tagged (MT) *vegt* to activate mesendoderm genes in animal cap explants cultured in solution containing cycloheximide (CHX) from stage 8 to stage 10.5 (Fig. S1D), but did not prevent the accumulation of the Myc-tagged VegT reporter protein (VegT-MT) (Fig. S1E), indicating that the potential target genes activated by ectopic ASCL1 might not be required for repression of VegT. Second, overexpression of *ascl1* did not affect the ability of Activin protein to induce Smad2/3 phosphorylation in animal explants (Fig. S1F) or the ability of *Xnr1* (also known as *nodal1*) to induce expression of mesendodermal genes (Fig. S1G). Importantly, coinjection of *Xnr1* partially restored expression of *sox17a*, *mixer* and *brachyury* (*bra*, also known as *T*) in the ASCL1-overexpressing gastrulae (Fig. 1H). We therefore conclude that ectopic ASCL1 inhibits the primary induction by maternal VegT and maternal Ascl1 might play a part to antagonize the function of VegT in mesendoderm formation.

### Increased expression of mesendoderm genes in Ascl1 morphants

**Fig. 2.**
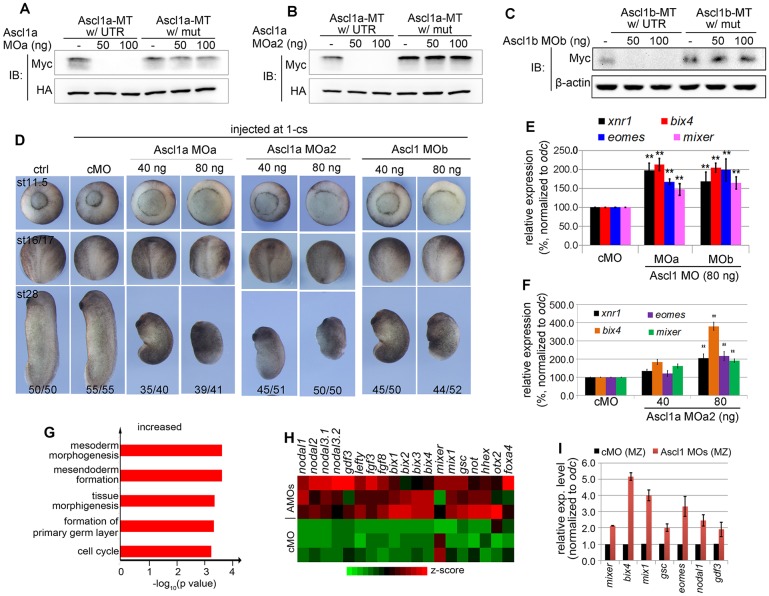
**Depletion of Ascl1 increases mesendoderm gene expression.** (A-C) Western blot analysis of the efficacies of Ascl1 MOa (A), MOa2 (B) and MOb (C) in blocking the translation of synthetic reporter *ascl1* mRNA with the wild-type 5′ UTR or with a mutated 5′ UTR. Doses of injected MOs: 50 or 100 ng. (D) Representative control and Ascl1 morphant embryos at different development stages. Experiment for each and every MO was repeated at least in three independent experiments, with embryos from one representative experiments shown in the figure. Numbers in the lower panels indicate incidence of morphological appearance resembling the embryos shown. (E,F) Control MO (cMO), Ascl1 MOa or MOb (E), or MOa2 (F) injected at one-cell stage and resultant morphants collected at stage 10.5 followed by qPCR examination of gene expression normalized to the levels of *odc*. **P<0.05, Student's *t*-test. (G) GO analysis of 384 upregulated genes in Ascl1 morphants against cMOs at stage 10.5-11. (H) Heat map presentation of the relative expression levels of a subset of VegT targets upregulated by Ascl1 depletion. (I) cMO or Ascl1 MOs (MOa+b) injected at one-cell stage and marginal zone explanted at the stage 8 and cultured to the equivalent stage 10.5 followed by qPCR examination of gene expression normalized to the expression levels of *odc*. Changes in expression of all examined genes relative to control are significant: Student's *t*-test, *P*<0.05.

In order to determine whether Ascl1 is a repressor of VegT function, we depleted Ascl1 in early *Xenopus* embryos. To do this, three translation-blocking morpholino oligos (MOs) were designed against both alleles of *Xenopus ascl1* gene – MOa and MOa2 for *ascl1a*, and MOb for *ascl1b* (Fig. S2A,B, Tables S1 and S2). Because a specific antibody against *Xenopus* Ascl1 is not available, we verified the efficacy of these MOs through sequential injections along with a reporter mRNA into fertilized eggs followed by western blot analysis of the epitope-tagged reporter protein. All three MOs (50-100 ng) efficiently blocked translation of their respective reporter mRNA containing the MO-complementary sequence, but not the translation of the MO-resistant ones (Fig. 2A-C, Fig. S2A,B). We then injected 40-80 ng of each MO alone into fertilized eggs at the one-cell stage and monitored these Ascl1 morphants during gastrula to tailbud stages. A 5 bp mismatch MO was also injected to control for the specificity of morpholino oligos, and did not cause appreciable abnormalities in the body pattern. By contrast, all three Ascl1 MOs caused a delay of gastrulation and neurulation, resulting in a body axis shortening in a dose-dependent manner by the early tailbud stage (Fig. 2D). qPCR analyses at stage 10.5 revealed that depletion of Ascl1, particularly under a higher dose of the MOs, significantly increased the expression of a subset of mesendoderm genes (Fig. 2E,F). It is worth noting that three different MOs exhibited similar effects in increasing mesendodermal genes, providing evidence that these MOs specifically deplete Ascl1. In addition, we noticed that depletion of Ascl1a or Ascl1b by the translation-blocking MOs (i.e. MOa and MOb) increased the expression of *ascl1a* and *ascl1b* at stage 10.5 for currently unknown reasons (Fig. S2C). It is possible that the degradation of maternal *ascl1* transcripts was somehow affected after injection of Ascl1 morpholino. It could also be due to increased expression of the zygotic *ascl1* in response to the depletion of maternal Ascl1. Regardless, injection of MOa or MOb alone was sufficient to increase the expression of mesendoderm genes (Fig. 2E). This could be explained, at least in part, by the observation that injection of Ascl1 MOa (MOb) could inhibit translation of the Ascl1b (1a) reporter (Fig. S2D). The observed cross-inhibition effects are likely to be due to the fact that 17 continuous nucleotides of MOa (MOb) are complementary to allele 1b (1a) with only one interrupting mismatch (Fig. S2B). Moreover, injecting a mix of MOa and MOb, at a suboptimal dose (30 ng) for each, resulted in similar gastrulation defects and body axis shortening as observed upon depletion of each allele alone (Fig. S2E, Fig. 2D). These observations collectively suggest that Ascl1a and Ascl1b play redundant roles in the regulation of mesendoderm formation.

**Fig. 3.**
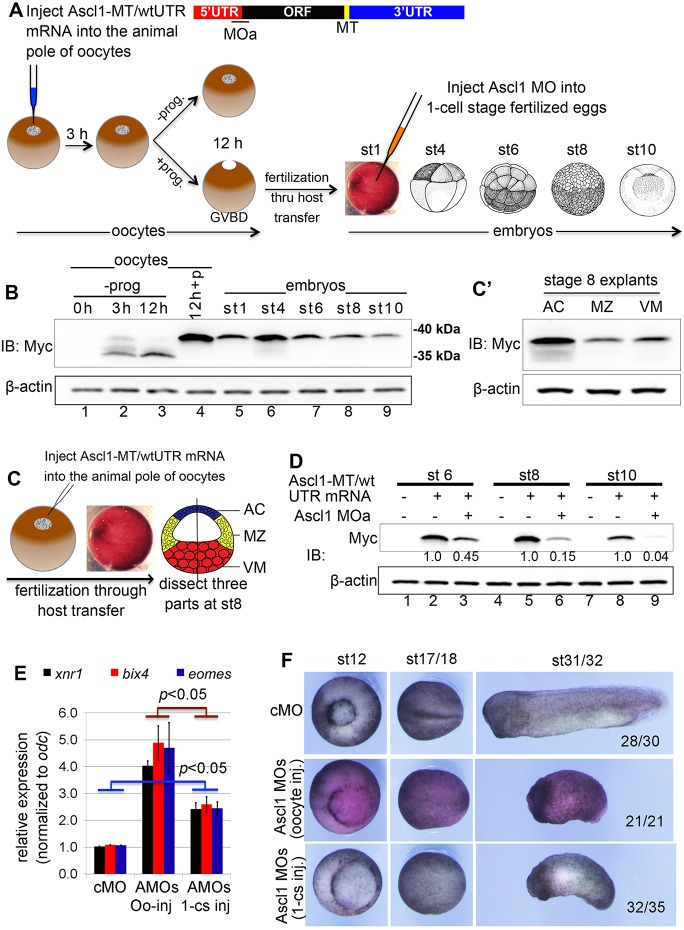
**Evidence for a maternal role for Ascl1 in regulating mesendoderm formation.** (A) Schematic of Ascl1-MT reporter construct and the experimental procedures for demonstrating the expression of Ascl1-MT reporter in oocytes and early embryos generated through host transfer. −prog., oocytes without progesterone treatment; +prog. or +p, oocytes treated with 2 µM progesterone. (B) Ascl1-MT reporter expressing oocytes or embryos frozen at the indicated time points after reporter mRNA injection into stage VI oocytes followed by western blotting using an anti-MT antibody. (C,C′) Embryos expressing Ascl1-MT reporter (30 each) at stage 8 separated into animal cap (AC), marginal zone (MZ) and vegetal mass (VM) and frozen immediately after separation followed by anti-MT western blotting. (D) Embryos expressing Ascl1-MT reporter injected with or without Ascl1 MOa at the one-cell stage cultured to stage 6-10 and frozen at the indicated time points followed by anti-MT western blotting. (E) qPCR comparison of gene expression in Ascl1 morphants produced from embryos expressing Ascl1-MT reporter injected with Ascl1 MOa before (oocyte-injection) or after (one-cell stage injection) fertilization. *P*-values obtained by performing a Student's *t-*test. (F) Embryos at different stages of development from experiments as described in E.

To gain a broader view of Ascl1 function in mesendoderm formation, we collected control and Ascl1 (MOa and MOb) morphants at stage 10.5 for RNA-seq analysis of gene expression (Tables S3 and S4). We found that Ascl1 depletion altered the expression of 1095 genes (*P*<10^−10^) (Fig. S2F). Near equal numbers of genes were increased (549 genes) or decreased (546 genes). Gene ontology (GO) analysis revealed that Ascl1 was required for the normal expression of those genes involved in gastrulation/embryonic morphogenesis (*P*<10^−4^, FDR<10%), mesoderm/primary germ layer formation (*P*<10^−4^), and anteroposterior pattern (*P*<10^−2^) (Fig. S2F). These results are consistent with the observed gastrulation and anteroposterior axis defects in Ascl1 morphants (Fig. 2D, Fig. 3F and Fig. S2E).

To better understand a repressor role for Ascl1, we focused on those genes that were increased twofold [log_2_ (fold change) ≥1 with *P*<10^−3^]. By these standards, we found 384 annotated genes (out of 952). GO analysis indicated that these 384 genes were primarily involved in formation/morphogenesis and primary germ layer formation (Fig. 2G). Additional analysis identified more than 20 known VegT targets that were significantly upregulated in Ascl1 morphants (Fig. 2H). We verified through qPCR in independent knockdown experiments that mesendoderm patterning genes, including members of TGF-β super family (*nodal1* and *gdf3*) and mix-like factors (*mixer*, *bix4*, *mix1*) were significantly increased in Ascl1-depleted marginal zone explants at the sibling stage 10.5 (Fig. 2I). These findings are consistent with the notion that expression of Ascl1 in the marginal zone is crucial for containing the VegT function around the marginal zone during the primary mesendoderm induction. Importantly, subsequent injection of MO-insensitive *ascl1* mRNA (100 pg) back into Ascl1 morphants partially rescued the expression of these genes (not shown), confirming that Ascl1 MOs specifically deplete Ascl1.

Because depletion of Ascl1 also affected expression of cell cycle regulators as revealed by GO analyses (Fig. 2G, Fig. S2F), we wanted to ensure that the control and Ascl1 morphants were compared for expression of mesendoderm genes at comparable stages of development. To do this, we microinjected Rhodamine-lysine dextran (RLDX) into one animal-pole cell at the 32-cell stage and counted the number of cells in the RLDX-labelled clone from the surface at stage 10.5. We found that the numbers of cells in the RLDX-labelled control MO clone and Ascl1 MOs clone were not significantly different (Fig. S3A,A′), suggesting that expression of the mesendoderm genes was compared between control and Ascl1 morphants at equivalent stages of development. Furthermore, using semi-quantitative PCR, we monitored expression of several mesendoderm genes in control and Ascl1 morphants during stage 9 to stage 11.5. The expression of these mesendoderm genes peaked at stage 10 and then gradually decreased during gastrulation in the controls (Fig. S3B, blue lines). Depletion of Ascl1 did not prevent these genes from being upregulated from stage 9-10; instead, sustained the expression of these genes at markedly higher levels during stage 10.5 to stage 11.5 (Fig. S3B, red lines), supporting the notion that depletion of Ascl1 probably has a direct effect on mesendoderm gene expression. However, additional studies are required to determine to what extent the deregulation of cell cycle regulators has contributed to the observed deregulation of mesendoderm genes in Ascl1 morphants. Based on these gain- and loss-of-function analyses, we conclude that Ascl1 acts as an essential repressor during mesendoderm formation, antagonizing VegT function in *Xenopus*.

### Evidence for Ascl1 as a maternal regulator of mesendoderm formation

The analyses presented above support an essential role for Ascl1 in regulating mesendoderm formation. However, it remains unclear whether the maternal Ascl1 is involved. We sought to provide evidence that Ascl1 protein is maternally stored. To do this, we generated an Ascl1-MT reporter construct that contains the endogenous 5′ and 3′ UTR of *Xenopus ascl1a* (named Ascl1-MT/wtUTR reporter) (Fig. 3A), aiming to demonstrate at least some aspects of maternal Ascl1 expression, particularly after meiotic maturation, when the maternal *ascl1* is released into cytoplasm after the germinal vesicle breakdown (GVBD). We injected the synthetic Ascl1-MT/wtUTR mRNA into the animal pole of full-grown oocytes. Leaving some of injected oocytes in culture without addition of progesterone, we stimulated the remainder with progesterone for meiotic maturation followed by fertilization through host transfer (Heasman, 2006). The reporter mRNA bearing oocytes and/or embryos (five each) from the host transfer at different developmental times were subsequently harvested to examine expression of the Ascl1-MT protein (Fig. 3A). Western blot analyses using an antibody against the Myc-tag epitope revealed interesting results, as shown in Fig. 3B. First, the Ascl1-MT reporter was translated more abundantly in the meiotic-matured oocytes than in those oocytes that were not treated with progesterone but cultured for the same period of time (Fig. 3B, lanes 3 and 4). In order to assess variations in reporter expression from oocyte to oocyte, we randomly picked six individual mRNA-injected oocytes after 12 h culture followed by western blot detection of Ascl1-MT protein. We found that the levels of reporter protein accumulation in these individual oocytes were comparable (not shown). Second, the migration rate of Ascl1-MT reporter protein in PAGE was reduced after meiotic maturation and in the subsequent stages of embryonic development (Fig. 3B, lanes 3-9). During meiotic maturation, the oocyte genome is transcriptionally quiescent and profound changes occur to maternal genes at post-transcriptional and/or post-translational levels. It has been reported that ASCL1 can be phosphorylated by kinase activities in *Xenopus* egg extracts (Ali et al., 2014). Although post-transcriptional/translational regulations of maternal *ascl1* are currently unknown and await detailed studies in future, our observations through an endogenous UTR-containing reporter argue that the maternal *ascl1* has a potential to be translated before fertilization.

Third, the reporter protein remained detectable up to the early gastrula stage, the latest time point we examined in these experiments (Fig. 3B, lane 9), supporting the notion that the maternally supplied Ascl1 protein is expressed at the right time for a role in the regulation of mesendoderm formation. It is worth noting that a trend of downregulation of the reporter protein during stage 6-10 was reproducibly observed in three independent experiments (Fig. 3B,D). Reporter protein expression did not display a simple and mono-toned change after fertilization through the early gastrula stage as representatively shown in Fig. 3B. Moreover, to examine the distribution of reporter protein in the mid-blastulae, we dissected the reporter mRNA bearing mid-blastulae into three parts along the animal vegetal axis (Fig. 3C). We found that the reporter protein was abundantly detected in primary ectoderm explants (ACs) and to a lesser extent in the marginal zone (MZ) and vegetal mass (VM) (Fig. 3C′), suggesting that the maternally supplied Ascl1 protein is also expressed in the correct place for a role in regulating mesendoderm formation.

We further exploited the reporter assay, aiming to assess whether post-fertilization injection of the MO effectively depletes maternal Ascl1 protein. Following the experimental design as briefly described above and schematically shown in Fig. 3A, we injected 80 ng Ascl1 MOa into the reporter bearing fertilized eggs at the one-cell stage, and harvested embryos at the later stages of development. We found that injection of Ascl1 MOa at the one-cell stage markedly inhibited the reporter protein accumulation at stage 6 (Fig. 3D, lanes 2 and 3). Greater reductions of reporter protein expression were observed at stage 8 and stage 10 (Fig. 3D, lanes 5 and 6, lanes 8 and 9). ASCL1 seems to be unstable a protein (Ali et al., 2014). The combinatorial effect of a MO blockade of translation and a rapid rate of protein turnover probably causes an effective knockdown of the maternally supplied Ascl1 during the blastula stages. We then tested whether injection of a mix of Ascl1 MOa and MOb into the one-cell stage embryos or into the full-grown oocytes differently affects mesendoderm gene expression. We found that the expression of several mesendoderm genes was significantly increased in the morphants at stage 10.5 produced by either method of MO injection (Fig. 3E), suggesting, by extrapolation from the reporter assay presented above, that injection of the MOs into fertilized eggs at the one-cell stage might have effectively diminished the function of maternal Ascl1. Perhaps it is not surprising that injection of the MOs into oocytes followed by host transfer increased the mesendoderm gene expression to a greater extent (Fig. 3E), if there is some maternal Ascl1 protein in eggs before fertilization. Our data thus argue for a maternal function for Ascl1 in the regulation of mesendoderm formation. During the later stages of development, Ascl1 morphants obtained through both methods of MO injection also demonstrated a delay of blastopore closure and neural tube closure, and a shortening of body axis (Fig. 3F).

### Ascl1 is required for neuralization of the prospective ectoderm

**Fig. 4.**
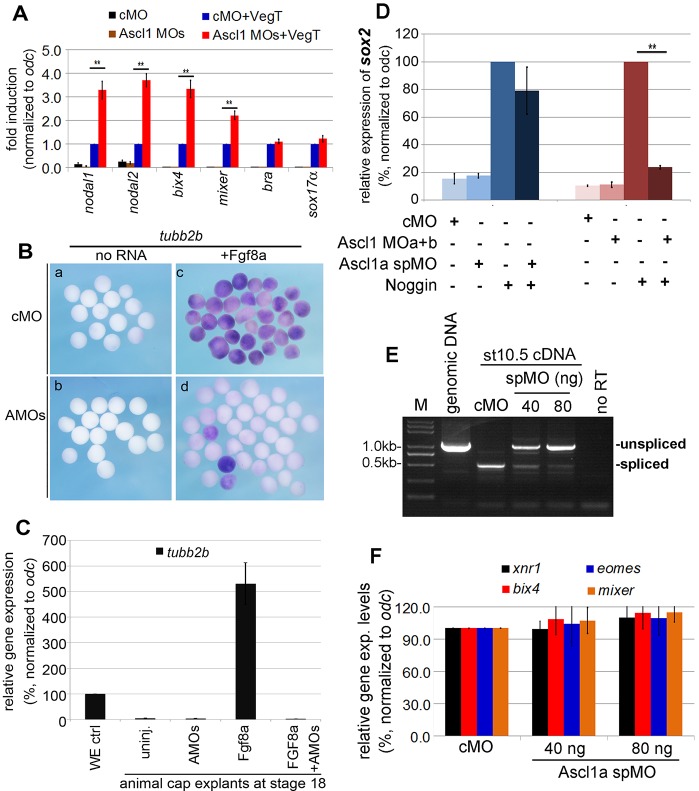
**Ascl1 is required for neurogenic potential in animal cells.** (A) qPCR comparison of mesendoderm gene expression (means±s.d.) in *vegt*-injected animal cap explants at sibling stage 10.5. Control and Ascl1 MOs (60 ng) were injected at one-cell stage. *vegt* mRNA (100 pg) was injected into animal pole at two-cell stage and animal caps were dissected at stage 8.5 and cultured to the sibling stage 10.5 for RT-qPCR analysis. **P<0.01. (B) Animal caps at the sibling stage 15 *in situ* hybridized with *tubb2b*. Control MO (cMO) and Ascl1 MOs (AMOs) (60 ng) were injected at one-cell stage, *fgf8a* mRNA (100 pg) was injected into animal pole at two-cell stage, and animal caps were dissected at stage 8.5 and cultured to the sibling stage 15 followed by *in situ* hybridization. (C) qPCR comparison of the expression of *tubb2b* (means±s.d.) in animal cap explants. The injection and dissection schemes were the same as described in B, except that the RT-qPCR analysis was performed at the sibling stage 18. (D) Animal cap explants injected with Ascl1 MOa+b or spMO isolated at stage 9 treated with or without Noggin protein (350 ng/ml) for 4 h, and then cultured to sibling stage 18 followed by qPCR detection of *sox2* expression (means±s.d.). **P<0.01. (E) RT-PCR verification of injection of Ascl1a spMO blocked *ascl1a* splice in stage 10.5 embryos. PCR using genomic DNA template also included as a control for the size of product amplified from unspliced mRNA/cDNA templates. (F) qPCR examination of gene expression in Ascl1a spMO at stage 10.5 (means±s.d.).

Our findings thus far predict that Ascl1 morphant animal explants should respond more strongly to ectopic VegT. To test this prediction, we compared the ability of VegT to induce mesendoderm genes in control and Ascl1 morphant explants, and confirmed the prediction (Fig. 4A). Because Ascl1 is essential for neurogenesis and its depletion in the prospective neural plate markedly inhibited activation of the β-tubulin *tubb2b* (Fig. S4A,B), we asked whether maternal Ascl1 was required for neural fate competence. To answer this, we examined expression of *tubb2b* in animal explants treated with the neural inducer Fgf8a (Fletcher et al., 2006). The activation of *tubb2b* by Fgf8a was clearly detectable using WISH (Fig. 4B) and qPCR (Fig. 4C) in control morphant explants. By contrast, this was not the case in Ascl1 morphant explants (Fig. 4B,C).

Proneural factors in the bHLH family are potent inducers of neuron formation in non-neural cells, but normal primary neurogenesis in *Xenopus* is accompanied with the neuralization of naive ectoderm by signals from the organizer. We therefore tested whether depletion of maternal Ascl1 affects the ability of Noggin protein to neuralize animal explants using the activation of *sox2* as an indicator. We found that depletion of Ascl1 significantly diminished the ability of Noggin to induce *sox2* expression in the animal explants at the equivalent stage 18 (Fig. 4D, mauve toned histograms).

To additionally address whether the function of Ascl1 in the regulation of neural fate competence is attributable to its maternal expression, we designed a splice-blocking MO (spMO) for Ascl1a according to the current *Xenopus laevis* genome annotation (Fig. S5A). The effectiveness of Ascl1a spMO was verified through a series of PCR analyses to block the splice of the single and only intron in the 3′UTR of *ascl1a* (Fig. 4E and Fig. S5B,C). Injection of the Ascl1a spMO at the one-cell stage did not cause a gastrulation delay, but did cause a delay in neurulation (Fig. S5D). qPCR examination at stage 18 indicated that expression of *tubb2b* was significantly reduced by Ascl1a spMO injection (Fig. S5E), confirming the relevance of Ascl1 for neurogenesis. Interestingly, however, the Ascl1a spMO did not exhibit a significant effect on the ability of Noggin protein to induce *sox2* expression (Fig. 4D, blue-toned bars), suggesting that any zygotic expression of *ascl1a* in the animal explants is dispensable for the neuralization activity of Noggin.

To assess whether depletion of the zygotic Ascl1 has an effect on mesendoderm formation, we examined mesendoderm gene expression in Ascl1a spMO-injected embryos. Curiously, we found that this was not the case (Fig. 4F). It is unknown at present whether the Ascl1a spMO cross-reacts with *ascl1b*; thus, it remains possible that the zygotic Ascl1b might have a role in mesendoderm formation. We therefore conclude that maternal Ascl1 is a crucial regulator of both mesendoderm and neural competence in naive ectoderm.

### An N-terminal domain is necessary and sufficient for ASCL1 to repress mesendoderm formation

**Fig. 5.**
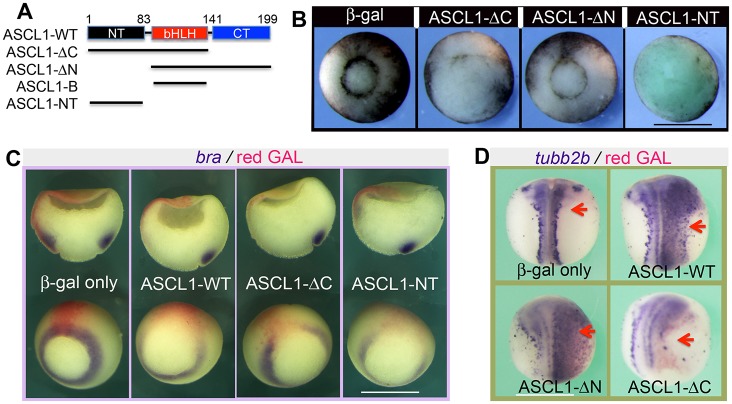
**ASCL1-NT is necessary and sufficient for inhibiting mesendoderm induction.** (A) Schematic depiction of serial-deletion mutants of ASCL1. (B) Vegetal view of representative embryos at stage 12. The indicated mRNAs were each individually injected into the vegetal pole at the two-cell stage. (C) Representative embryos (bottom row) and bisections (top row) at stage 11 stained with Red-Gal and *in situ* hybridized for *bra*. (D) Representative embryos at stage 16 stained with Red-Gal and *in situ* hybridized for *tubb2b*. Arrows indicate the injected sides. Scale bars: 1 mm.

The bHLH and C-terminal domains of ASCL1 are involved in the transactivating capacity of Ascl1 (Henke et al., 2009; Johnson et al., 1992; Sugimori et al., 2008). To determine which part of the protein is essential for the repressor activity of ASCL1, we generated three ASCL1 deletion mutants: ASCL1-ΔN (lacking the N-terminal sequence upstream of the basic motif), ASCL1-ΔC (lacking the CT) and ASCL1-NT (lacking both the bHLH and CT domains) (Fig. 5A). Each and every of these mutants were then examined through the ectopic expression and by the criteria of their abilities to affect blastopore formation and/or gene expression. Results from these assays indicate that ASCL1-NT is both necessary and sufficient for ASCL1 to inhibit blastopore formation (Fig. 5B) and expression of *bra* (Fig. 5C). Instead, the C-terminal part is dispensable in both respects (Fig. 5B,C).

In addition, we found that ASCL1-ΔN was able to induce ectopic *tubb2b* expression in non-neural ectoderm (Fig. 5D), whereas ASCL1-ΔC appeared to be a loss-of-function mutant in this regard (Fig. 5D). Overexpression of ASCL1-NT in the animal pole did not discernibly affect *tubb2b* expression (data not shown).

These structure-function analyses indicate that the N-terminal domain is crucial for ASCL1 to repress VegT but dispensable for its proneural activity under the current conditions. Few studies in the literature have examined the N-terminal domain of ASCL1. The present findings prompted us to further analyse how this domain might permit ASCL1 to function as a repressor.

### HDAC1 is associated with ASCL1 in regulating mesendoderm formation

In order to better understand how ASCL1 acts as a repressor, we overexpressed Myc-tagged ASCL1-ΔC in the early embryos and performed an anti-Myc ChIP analysis, where the ChIP lysate was subjected to a mass spectrometric identification of proteins associated with MT-ASCL1-ΔC. This led us to focus on HDAC1 because it was among the most enriched in the MT-ASCL1-ΔC chromatin fragments pulled down by the anti-MT antibody (data not shown). We then performed a series of experiments to verify the functional relevance of HDAC1 in ASCL1 repression of mesendoderm formation.

**Fig. 6.**
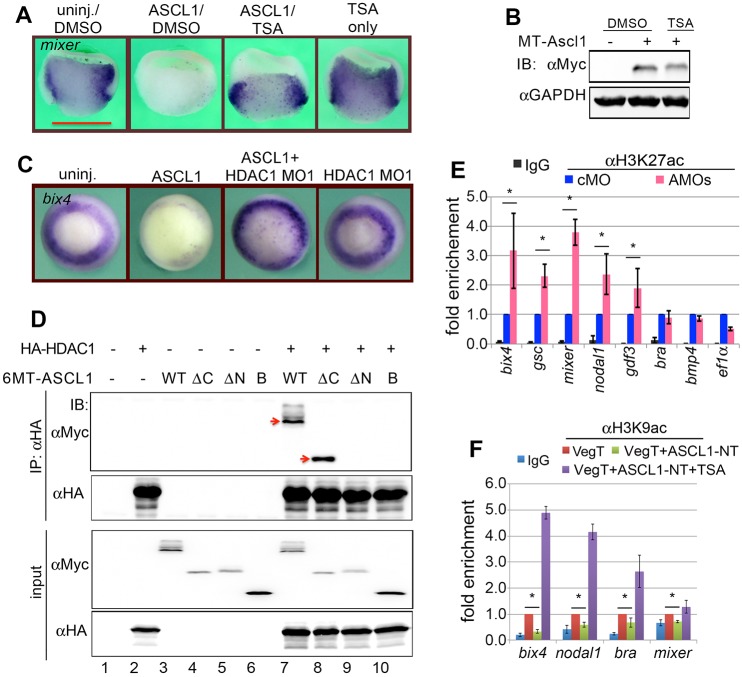
**HDAC1 is associated with ASCL1 in regulating mesendoderm formation.** (A) Bisection view of embryos showing that the expression of *mixer* was inhibited by ASCL1 overexpression and rescued by the application of TSA (100 nM). (B) Anti-MT western blot showing the expression of MT-ASCL1 in the absence and presence of TSA treatment (100 nM). (C) HDAC1 MO1 rescues *bix4* expression in ASCL1-overexpressing embryos at stage 10.5. (D) Results from co-immunoprecipitation (CoIP) using anti-HA antibody followed by western blot analysis using anti-MT antibodies. Red arrows indicate that ASCL1-WT (lane 7) and ASCL1-DC (lane 8) were brought down by anti-HA antibody. (E) ChIP-qPCR showing that the depletion of maternal Ascl1 increases H3K27ac at the promoter regions of VegT targets. Values are means±s.d. *P<0.05. (F) ChIP-qPCR showing that ASCL1-NT reduces H3K9ac marks at the promoter regions of VegT targets in an HDAC activity-dependent manner. Values are means±s.d. *P<0.05. TSA: 100 nM.

First, we utilized trichostatin (TSA) and other small molecular inhibitors of HDACs to test whether HDAC activity is required for Ascl1 repression of mesendoderm genes. We found that TSA cancelled out the effects of ectopic ASCL1 in repressing mesendoderm gene expression (Fig. 6A). The dose of TSA was carefully titrated such that its application did not cause abnormal gene expression (Fig. S6A,B) or affect the expression of ectopic ASCL1 protein (Fig. 6B). We also tested MS-275 (Entinostat) and valproic acid (VPA) (Fig. S6C-E), and reached the same conclusion that HDAC activity is required for ASCL1 to repress mesendoderm gene expression.

Second, we depleted HDAC1 using two non-overlapping translation-blocking MOs (Fig. S7A,B). qPCR analyses revealed that injection of either HDAC1 MO1 or MO2 significantly increased the expression of a subset of mesendoderm genes (Fig. S7C), suggesting a role for HDAC1 in controlling mesendoderm formation. Depletion of HDAC2 using a MO did not increase mesendoderm gene expression (Fig. S7A-C). Importantly, injection of HDAC1 MO1 partially restored *bix4* expression in ASCL1-overexpressing embryos (Fig. 6C), indicating that HDAC1 is a candidate co-repressor for ASCL1 in controlling mesendoderm formation.

Third, we performed coimmunoprecipitation experiments using 293T cell lysate overexpressing the relevant 6MT-ASCL1 or HA-HDAC1 constructs. Both 6MT-ASCL1-WT and 6MT-ASCL1-ΔC were readily detectable in the immunocomplexes brought down by anti-HA antibodies (Fig. 6D, red arrows), indicating that the CT is not necessary for this association. This finding was confirmed by tag-swap experiments followed by coimmunoprecipitation using anti-Myc antibodies (Fig. S6F). In striking contrast, deletion of the N-terminal domain (NT) as in ASCL1-ΔN and/or ASCL1-B completely abolished the association between ASCL1 and HDAC1 (Fig. 6D, lanes 9 and 10), revealing that the NT is required for ASCL1 to interact with HDAC1.

Lastly, we performed ChIP-qPCR to detect H3K27 acetylation (H3K27ac), a hallmark associated with the promoters of actively transcribed genes, at several VegT target genes after Ascl1 depletion by injecting a mix of MOa and MOb at the one-cell stage. We found that depletion of Ascl1 resulted in a ∼twofold increase of the H3K27ac levels at the promoter regions of *bix4*, *mixer*, *gsc*, *nodal1*, *gdf3* (Fig. 6E). Additional ChIP-qPCR analysis indicated that H3K9ac was also increased at the same set of mesendoderm genes by Ascl1 depletion (not shown), indicating that Ascl1 is able to modulate both H3K9ac and H3K27ac marks at mesendoderm genes that are controlled by VegT. By contrast, ectopic ASCL1 resulted in a marked reduction of H3K27ac and H3K9ac induced by microinjected VegT (not shown), corroborating the findings from the functional analyses that Ascl1 is a repressor of VegT. Importantly, ASCL1-NT, which lacks other known functional domains, was also observed to inhibit ectopic VegT-induced H3K9ac (Fig. 6F). Moreover, application of TSA cancelled the repressive activity of ASCL1-NT against VegT as revealed through ChIP-qPCR (Fig. 6F), confirming the sufficiency of the N-terminal sequence in repressing mesendoderm gene expression.

Taken together, we conclude that the repressor activity of Ascl1 against VegT is attributable to the N-terminal domain-mediated recruitment of HDAC activity. Our current loss-of-function analyses for HDAC1 indicate that HDAC1 is critically required for Ascl1 to antagonize VegT function in mesendoderm formation.

### ASCL1 is associated with VegT and mesendoderm genes

**Fig. 7.**
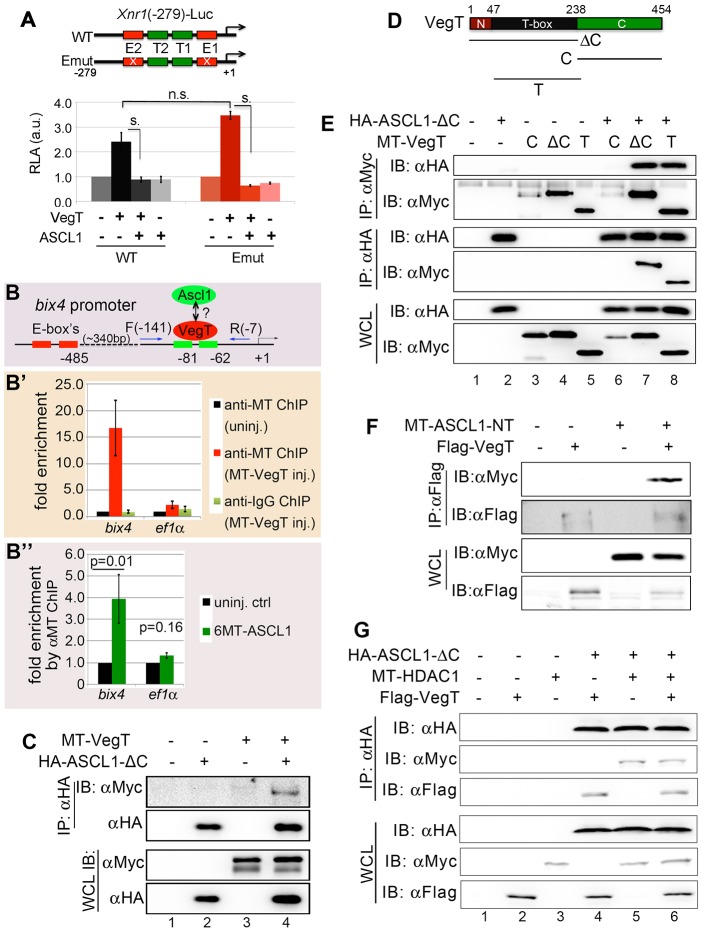
**Ascl1 is associated with VegT and mesendoderm genes.** (A) Luciferase reporter assay shows that mutating the E-boxes in the proximal regulatory region of *Xnr1* does not affect Ascl1 repression of the promoter activity induced by VegT. T1 and T2: functional T-boxes in the minimal *Xnr1* promoter. E1 and E2: two putative E-boxes found in the *Xnr1* minimal promoter by MEME algorithm. Crossed red rectangles indicate mutated E-boxes. s., significantly different (*P*<0.01). (B) Schematic of a minimal *bix4* promoter with functionally characterized T-boxes and putative E-boxes highlighted. Green rectangles: T-boxes; red rectangles: putative E-boxes. 340 bp indicates the distance from the 3′ end of the E-boxes upstream of the 5′ end of the T-boxes. F, forward primer; R, reverse primer. (B′) ChIP-PCR results showing anti-MT-VegT ChIP recovers a *bix4* promoter fragment from the T-box-containing region. (B″) ChIP-PCR results showing anti-MT-ASCL1 ChIP recovers a *bix4* promoter fragment from the T-box-containing region. Values are means±s.d. (C) Western blots of anti-HA immunocomplexes detected by anti-Myc-tag or anti-HA antibodies indicating the association between ASCL1-ΔC and VegT. (D) A schematic depiction of MT-VegT serial deletions used in the CoIP/western blotting experiments shown in E,F. (E) HA-ASCL1-ΔC interacts with the T-box domain of VegT. (F) MT-ASCL1-NT interacts with Flag-VegT. (G) Results from CoIP followed by western blot analyses showing HDAC1 and VegT non-mutual exclusively associated with ASCL1.

The association between ASCL1 and its cognate E-box motif is required for its activator function. Our results thus far strongly suggested that Ascl1 represses mesendoderm gene expression independent of this kind of association (Fig. S1C, Fig. 5B,C). To confirm this notion, we made use of a luciferase reporter gene driven by a minimal promoter of *Xnr1* (*nodal1*), which has been previously characterized by others (Casey et al., 1999; Tada et al., 1998). This *Xnr1* minimal promoter contains two functional T-box motifs (Casey et al., 1999; Tada et al., 1998), which are closely flanked by a putative E-box on each side, as revealed by MEME algorithm (Fig. 7A). We observed that the ectopic VegT*-*induced *Xnr1*-Luc reporter activities were significantly inhibited by coinjection of ASCL1, regardless of whether the E-boxes were intact or mutated (Fig. 7A), suggesting that these E-boxes are not required for ASCL1 to repress mesendoderm gene expression. We then examined whether ASCL1 is associated with a mesendoderm gene possibly through VegT. To this goal, we overexpressed MT-ASCL1 and performed ChIP-qPCR to recover a promoter fragment of *bix4* (Fig. 7B), in which two functional T-boxes have been identified within 100 bp upstream of the transcriptional start site (Casey et al., 1999; Tada et al., 1998). We verified through anti-MT ChIP followed by qPCR that the overexpressed MT-VegT was associated with these functional T-boxes in the *bix4* promoter (Fig. 7B′). More importantly, anti-MT ChIP using lysate overexpressing 6MT-ASCL1 but not MT-VegT also recovered the same promoter fragment of *bix4* (Fig. 7B″). Note that the nearest degenerate E-boxes are located 300 bp upstream of the two functional T-boxes in the *bix4* promoter, which allowed us to design qPCR primers that would not amplify the E-box-containing sequences. The results suggested that ASCL1 is associated with VegT targets.

To demonstrate the interaction between ASCL1 and VegT, we performed CoIP experiments and found that MT-VegT and HA-ASCL1-ΔC were detected in the same immunocomplexes (Fig. 7C, lane 4). To determine which domain of VegT is involved in this interaction, we used serial deletion mutants for VegT as schematically depicted in Fig. 7D. Coimmunoprecipitation revealed that ASCL1-ΔC interacted with VegT-ΔC and VegT-T mutants (Fig. 7E, lanes 6 and 8). More strikingly, the N-terminal domain alone was also able to interact with VegT (Fig. 7F, far right lane). Lastly, we found that addition of HDAC1 did not prevent ASCL1 from associating with VegT, and VegT did not prevent ASCL1 from associating with HDAC1 in the transfected 293T cell lysate (Fig. 7G), suggesting that the binding of HDAC1 and VegT with ASCL1 might not be mutually exclusive.

Taken together, we conclude that ASCL1 represses VegT at the regulatory regions of mesendoderm genes depending on the HDAC activity represented by HDAC1.

## DISCUSSION

The function of Ascl1 and Ascl3 in *Xenopus* has been extensively studied through ectopic expression analysis of its proneural function (Ali et al., 2014; Ferreiro et al., 1994; Perron et al., 1999; Turner and Weintraub, 1994; Zimmerman et al., 1993), whereas few loss-of-function studies have been devoted to Ascl1 during peripheral nervous system development, particularly in the specification of anteroventral noradrenergic neurons (Parlier et al., 2008; Wylie et al., 2015). In this study, we found evidence, through both gain- and loss-of-function approaches, that Ascl1 regulates early development, well before neurogenesis, by repressing mesendoderm induction by VegT in *Xenopus*.

ASCL1 has been shown to primarily function as a transcriptional activator during the conversion of non-neural somatic cells into neurons (Raposo et al., 2015; Wapinski et al., 2013). It has also been shown that ASCL1 positively regulates a large number of genes during spinal neurogenesis in mouse embryos (Borromeo et al., 2014). The transactivating activity of ASCL1 requires basic domain-mediated DNA binding, and both the HLH domain and the C-terminal sequence downstream of the bHLH positively regulate ASCL1 transactivating activities (Johnson et al., 1992). The function of the N-terminal sequence of ASCL1 is less well understood. In this study, we provide evidence that ASCL1 represses mesendoderm induction solely depending on the ability of its N-terminal sequence to interact with HDAC1 and VegT probably in a non-mutually exclusive fashion. These findings ascribe a novel function for the N-terminal domain of ASCL1 in regulating mesendoderm formation in *Xenopus*. HDAC activity is essential for ASCL1 repressor function during mesendoderm induction. Intriguingly, although both HDAC1 and HDAC2 are abundantly expressed in *Xenopus* eggs, our current analyses indicate that HDAC1, but not HDAC2, is crucially required for ASCL1 to antagonize VegT function. We therefore hypothesize that Ascl1 recruits HDAC1 to the proximity of VegT-controlled mesendoderm genes. HDAC1, in turn, creates a chromatin environment in which it is unfavourable for VegT to act.

We found that Ascl1 inhibits mesendoderm induction by VegT but not by Nodal/Activin. Oct3/4 homologs in *Xenopus* have been previously shown to inhibit mesendoderm formation (Cao et al., 2007, 2006, 2008). As stem factors in *Xenopus*, POU-V proteins (Oct60 and Oct25, in particular) also inhibit the activity of signalling through Nodal/Activin and Wnt, in addition to VegT (Cao et al., 2007, 2006, 2008). Furthermore, we found that Ascl1 is required for responsiveness to Fgf8a in neural induction, resembling another additional Oct3/4 homolog in *Xenopus*, i.e. Oct91. Oct91 is important for the timing of competence transition from mesoderm to neural cell fates (Snir et al., 2006). Our RNA-seq and additional qPCR analyses did not find a significant alteration of POU-V gene expression upon depletion of Ascl1. Perhaps, Ascl1 functions in parallel to or together with POU-V in determining the FGF competence in the prospective ectoderm during neural fate commitment. The maternal deposition of *ascl1*, and possibly Ascl1 in the animal pole may fine-tune the VegT mesendoderm responsiveness around the marginal zone on the one hand and prepattern neural fate in the animal hemisphere on the other. Recent studies in induced neuron formation *in vitro* have started to provide insights into how ASCL1 functions as a prepatterning factor (Raposo et al., 2015; Wapinski et al., 2013). We speculate that pre-neurula expression of ASCL1 at least plays a part in the timely activation of neuronal genes such as *tubb2b* during early neurula stages. Further study is needed to better understand how the pre-neurula expression of Ascl1 functions as a transactivator and promotes neurogenesis.

It remains unclear whether or how much Ascl1 protein is maternally stored. We have previously shown that maternal *mga* (*max-gene associated*) is stored in germinal vesicle and plays a role in the formation of the dorsal axis through Wnt signalling (Gu et al., 2012). Here, we show that maternal *ascl1* is also localized to GVs. Interestingly, it has been recently reported that a large set of spliced intronic RNAs are stored in GVs of *Xenopus tropicalis* oocytes and transmitted to the early developmental embryos (Gardner et al., 2012). Thus, entrapment of RNAs in GVs might represent a novel type of maternal gene localization mechanism. The invariant animal pole positioning of the GV provides a spatial control mechanism of the entrapped maternal RNAs through a delayed nuclear export (Gardner et al., 2012). Our current findings using an *ascl1a* UTR-containing reporter suggest that Ascl1 protein is produced during and after meiotic maturation. Injection of translation-blocking MOs either before or after fertilization, but not of a splice-blocking MO, against Ascl1 unveils a de-repression effect on VegT target expression, arguing for a maternal role for Ascl1 in the regulation of mesendoderm formation in *Xenopus*. Both *ascl1* mRNA and Ascl1 reporter protein are detectable in the marginal zone of blastulae, where VegT transiently activates Nodal-related growth factors and Mix-like proteins during gastrulation. Ascl1 depletion results in increased and/or sustained expression of these patterning genes, thus perturbing the progression of gastrulation.

One additional aspect of our current observations that is not accounted for is a potential role for cell cycle regulators in Ascl1 morphants (Fig. 2G, Fig. S2F). During neurogenesis, the function of Ascl1 is crucial for both the proliferation of neural progenitors and the post-mitotic differentiation of neurons (reviewed in Castro and Guillemot, 2011; Hardwick et al., 2014). It is conceivable that the function of Ascl1 in *Xenopus* is regulated in a cell-cycle progression-coordinated fashion during blastula to gastrula stages, when embryonic cells face a choice of dividing and/or differentiating. More studies are needed in future to unravel the function of the multifaceted cell fate regulator Ascl1.

## MATERIALS AND METHODS

### Embryos, explants and small-molecule inhibitors

*Xenopus* eggs and embryos were staged and handled according to standard protocols (Nieuwkoop and Faber, 1994; Sive et al., 2000). Synthetic mRNAs or MOs were microinjected into embryos cultured in 2% Ficoll 400 in 0.3× MMR (1× MMR: 100 mM NaCl, 2 mM KCl, 2 mM CaCl_2_, 1 mM MgCl_2_) at desired stages, as specified in the text and the figure legends. Isolation of animal cap explants was carried out with embryos cultured in 1× MMR at stage 8.5 using a pair of watchmaker's forceps. All HDAC inhibitors (HDACIs) were dissolved in DMSO and used at doses as indicated in the legends for Fig. 6 and Fig. S5.

### mRNA synthesis and DIG-UTP probe labelling

Information regarding the cDNA constructs and enzymes used for linearization and *in vitro* transcription using a mMessage mMachine SP6 Kit and for DIG-UTP probe labelling using a Roche T7 polymerase kit can be found in Table S7.

### Whole-mount *in situ* hybridization

Whole-mount *in situ* hybridization (WISH) was performed as previously described (Ding et al., 2013; Zhang et al., 2014). When β-galactosidase was included as a lineage tracer, Red-Gal staining was performed before the *in situ* procedures.

### qPCR

qPCR was performed through procedures as described previously (Ding et al., 2013; Zhang et al., 2014). All qPCR results are presented as the relative expression level or fold induction compared with the control embryos/explants; the expression levels in the control embryos/explants were set at 100%. *odc* was used a loading control. Primer sequence information can be found in Table S6.

### ChIP analysis and RNA-seq

ChIP-qPCR was performed as described previously (Blythe et al., 2009). The sonication protocol was optimized to obtain approximately 400-500 bp genomic DNA fragment smears on agarose gels. Information regarding the ChIP primer sequences and RNA-seq data analysis can be found in Tables S3, S4 and the supplementary Materials and Methods. The RNA-seq data are available at GEO with accession number GSE76915.

### Coimmunoprecipitation and western blotting

Coimmunoprecipitation (CoIP) and western blotting were performed as described previously (Ding et al., 2013; Zhang et al., 2014). HDAC1 was cloned into the pCS107 vector using *Xenopus* cDNA. Information regarding the antibodies used in this study can be found in Table S5 and the supplementary Materials and Methods. All constructs are available upon request (see Table S7).
